# Efficient isolated microspore culture protocol for callus induction and plantlet regeneration in *japonica* rice (*Oryza sativa* L.)

**DOI:** 10.1186/s13007-024-01189-0

**Published:** 2024-05-24

**Authors:** Runhong Gao, Yingjie Zong, Shuwei Zhang, Guimei Guo, Wenqi Zhang, Zhiwei Chen, Ruiju Lu, Chenghong Liu, Yifei Wang, Yingbo Li

**Affiliations:** Shanghai Key Laboratory of Agricultural Genetics and Breeding, Biotechnology Research Institute, Shanghai Academy of Agricultural Sciences, Shanghai, 201106 China

**Keywords:** Isolated microspore culture, *Oryza sativa* L., Low-temperature pre-treatment, Microspore developmental stage, Callus induction, Plantlet regeneration

## Abstract

**Background:**

Isolated microspore culture is a useful biotechnological technique applied in modern plant breeding programs as it can produce doubled haploid (DH) plants and accelerate the development of new varieties. Furthermore, as a single-cell culture technique, the isolated microspore culture provides an excellent platform for studying microspore embryogenesis. However, the reports on isolated microspore culture are rather limited in rice due to the low callus induction rate, poor regeneration capability, and high genotypic dependency. The present study developed an effective isolated microspore culture protocol for high-frequency androgenesis in four *japonica* rice genotypes. Several factors affecting the isolated microspore culture were studied to evaluate their effects on callus induction and plantlet regeneration.

**Results:**

Low-temperature pre-treatment at 4 ℃ for 10–15 days could effectively promote microspore embryogenesis in *japonica* rice. A simple and efficient method was proposed for identifying the microspore developmental stage. The anthers in yellow-green florets located on the second type of primary branch on the rice panicle were found to be the optimal stage for isolated microspore culture. The most effective induction media for callus induction were IM2 and IM3, depending on the genotype. The optimal concentration of 2, 4-D in the medium for callus induction was 1 mg/L. Callus induction was negatively affected by a high concentration of KT over 1.5 mg/L. The differentiation medium suitable for *japonica* rice microspore callus comprised 1/2 MS, 2 mg/L 6-BA, 0.5 mg/L NAA, 30 g/L sucrose, and 6 g/L agar. The regeneration frequency of the four genotypes ranged from 61–211 green plantlets per 100 mg calli, with Chongxiangjing showing the highest regeneration frequency.

**Conclusions:**

This study presented an efficient protocol for improved callus induction and green plantlet regeneration in *japonica* rice via isolated microspore culture, which could provide valuable support for rice breeding and genetic research.

## Background

Rice (*Oryza sativa* L.) (2n = 2x = 24) is one of the most important food crops in the world. The development of high-yield rice is crucial for meeting the continuously growing food needs of the world’s population [1]. The Green Revolution and hybrid technology have resulted in significant yield advancements for rice. However, following the remarkable success of these two breakthroughs, rice output levels have nearly plateaued [2]. Novel gene sources and innovative breeding selection procedures are urgently needed to develop high-yielding rice varieties to address population increase and climate change issues [2, 3]. Conventional breeding methods based on hybridization and phenotypic selection are time-consuming [4]. Biotechnologies, particularly double haploid (DH) technology that can produce homozygous lines within a short time (one “step” versus several selfing generations), provide a powerful tool for plant breeding [5, 6]. Moreover, DHs are crucial in applied research such as recombination and fixation of genetic variance, mapping, genomic selection and genomic prediction, genetic transformation, mutation, genome editing, epigenetics, and reverse breeding [6, 7].

Isolated microspore culture and anther culture are the most used and efficient ways to produce DH plants by androgenesis. Although anther culture is well exploited in *japonica* rice breeding [8–10], isolated microspore culture is superior to anther culture in a number of ways. The isolated microspore culture system can produce large numbers of uniformly treated microspores that develop rapidly into a mass of embryos, with many differentiating into regenerated plantlets. This system can provide better nutrition for the developing microspores. Microspores are released from the anthers through mechanical crushing, while the anther walls are removed by filtration, thus avoiding the production of diploid embryos and plants from somatic anther tissue. Isolated microspore culture is an efficient method for monitoring and researching microspore embryogenesis. The transition from a single cell to a multicellular structure and the subsequent production of callus can be easily monitored microscopically [11–14].

The low callus induction rate, poor regeneration capability, and high genotypic dependency have limited studies on DH production by isolated microspore culture in rice despite the benefits of this technique [15, 16]. Efficient isolated microspore culture is the basis for the successful application of this technique in breeding programs and other genetic studies [17]. Many factors affect the efficiency of isolated microspore culture in rice, including the genotype, microspore developmental stage, pre-treatment, medium composition, and growth regulators [15, 16, 18, 19]. The *japonica* varieties are generally more responsive to microspore embryogenesis than the *indica* varieties [8]. Combining the excellent characteristics of *indica* rice with the good androgenic response of *japonica* rice may improve the response of microspore embryogenesis [8, 20, 21]. The microspores at the mid- to late-uninucleate stage are best for embryogenic induction. The embryogenic response can be improved through the precise identification of this stage [22]. Cold pre-treatment has been reported to promote the microspore shift from gametophytic to sporophytic and guide the continuous microspores division followed by formation of the callus or embryo. However, the optimal duration of the cold pre-treatment may vary depending on the species and even the variety [23]. Medium composition and growth regulators also play important roles in microspore embryogenesis, which may determine whether the androgenesis would be initiated or not [16]. Therefore, the optimization of these key factors is necessary for the establishment of an efficient isolated microspore culture system for selected *japonica* rice varieties.

This study aimed to develop an effective approach for isolated microspore culture in *japonica* rice. The effects of various factors, including cold pre-treatment, microspore developmental stage, induction and differentiation media, and plant growth regulators, on the enhancement of callus initiation and plantlet regeneration in *japonica* rice through isolated microspore culture were evaluated.

## Materials and methods

### Plant materials

Four varieties of rice, Nipponbare, Zhonghua11 (ZH11), Nanjing46 (NJ46), and Chongxiangjing (CXJ), were used in this study. Among these, Nipponbare and ZH11 are commonly utilized as model *japonica* rice varieties, while NJ46 and CXJ are new improved *japonica* rice varieties provided by the CAS Center of Excellence in Molecular Plant Sciences in Shanghai, China. These donor plants were cultivated on the farm of the Shanghai Academy of Agricultural Sciences in Shanghai, China. Rice panicles were collected between 9:00–10:00 am when the flag leaf sheath or boot was swollen during the booting stage.

All chemicals and reagents used in this study were purchased from Sigma-Aldrich (St Louis, MO, USA) unless otherwise specified.

### Low-temperature pre-treatment

The rice panicles were collected and after removal of the leaves, were wrapped in moist gauze, placed in a plastic bag, and refrigerated at 4 ℃ (HYC-650, Haier Biomedical, Qingdao, China). For the determination of microspore viability and developmental stage, the panicles were refrigerated at 4 ℃ for 10 days. To identify the optimal duration of refrigeration, the panicles were maintained for 7, 10, and 15 d at 4 ℃. Panicles without low-temperature pre-treatment (0 d) were designated as controls. Twelve days of low-temperature pre-treatment at 4 ℃ was chosen for the remaining isolated microspore culture experiments.

### Primary branch classification, microspore viability assessment and microspore developmental stage identification

**Fig. 1:**
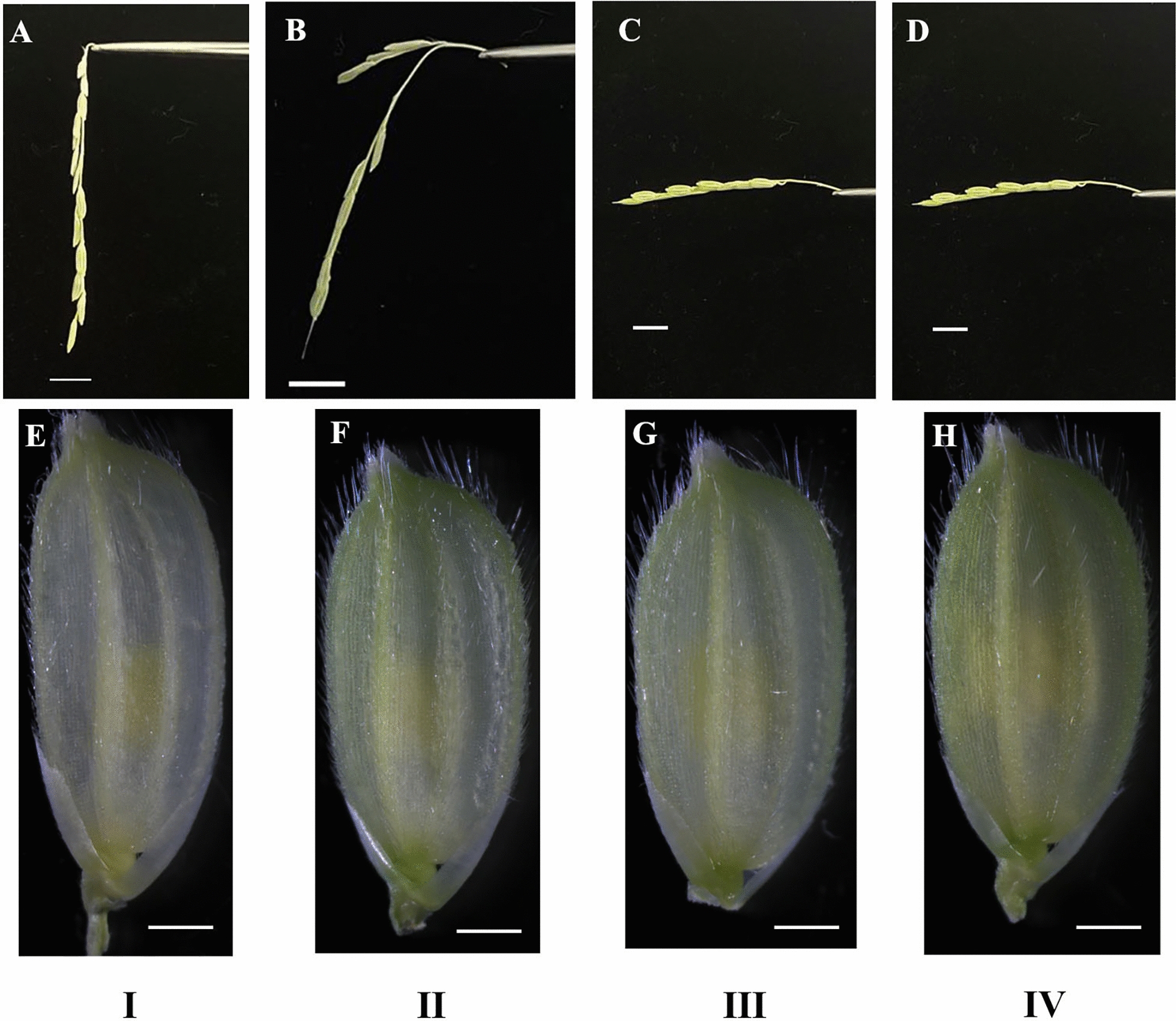
The curvature of the primary branch (**A**, **B**, **C**, **D**, bar = 1 cm) and the color of the floret (**E**, **F**, **G**, **H**, bar = 1 mm) classification. Type I: The primary branch is perpendicular to the ground, equal to 90° (**A**), with light yellow floret color (**E**); Type II: The angle between the primary branch and the ground is greater than 90° and less than 180° (**B**), with yellow-green floret color (**F**); Type III: The primary branch is parallel to the ground, equal to 180° (**C**), with yellow-green floret color (**G**); Type IV: The primary branch is parallel to the ground, equal to 180° (**D**), with green floret color (**H**)

The panicle was carefully peeled from the leaf sheath. The primary branch was severed from the panicle and classified into four types based on the curvature of the primary branch and the color of the floret. The curvature of the primary branch was classified as being perpendicular to the ground, parallel to the ground, or at an intermediate angle. The color of the floret was either light yellow, yellow-green, or green. Types I to IV were defined as shown in Fig. 1.

Microspore viability was determined using the fluorescein diacetate (FDA) staining method reported by Lu et al. [24]. Microspore suspension and FDA dye solution were mixed evenly in a 1:1 ratio, kept at 25 ℃ in the dark for 10 min, and then evaluated under a fluorescence microscope (EVOS FL Auto2, Thermo Fisher Scientific, Waltham, MA, USA). Microspores with green fluorescence were the viable microspores. Microspore viability was calculated by dividing the number of viable microspores in the dark field by the total number of microspores detected in the bright field.

The microspore developmental stage was determined using the 4',6-diamidino-2-phenylindole (DAPI) staining method described by Bhatia et al. [17], with slight modifications. The microspores were treated with Carnoy's Fluid (alcohol: acetic acid = 3:1) for 10 min to fix them. After discarding the supernatant, 10 μl of double-distilled water and 10 μl of 0.1 mg/mL DAPI staining solution were added, mixed thoroughly, and incubated at 25 ℃ in the dark for 10 min. The sample was then observed under fluorescence microscopy as described above.

### Microspore isolation and culture

Microspores were isolated and cultured as described by Lu et al. [24]. Following cold pre-treatment, the panicles were surface-sterilized in a 10% (v/v) NaOCl solution (Sinopharm Chemical Reagent, Shanghai, China) for 10 min and rinsed five times with sterile water. The anthers were carefully collected using tweezers and transferred into a 50 mL centrifuge tube in a sterile environment. The anthers from about 100 florets were placed into each tube, and 12 mL of the extraction solution was added. The extraction solution contained 60 g/L mannitol, 1.1 g/L CaCl_2_, and 0.976 g/L 2-(N-morpholino) ethane sulfonic acid hydrate (MES), pH 5.8, and was then sterilized by filter-sterilization (0.22 μm PES, Merck Millipore, Darmstadt, Germany). The anthers were homogenized three times using a high-speed homogenizer (D-500D, Wiggens, Wuppertal, Germany) at speed of 3000 rpm for 5 s per time, and the resulting suspension was filtered through a metal mesh with a pore size of 48 μm (Hongxiang, Shanghai, China). The microspores were collected by centrifugation (TD5M, Bioridge, Shanghai, China) at 500 rpm for 5 min.

The isolated microspores were incubated in the extraction solution at 25 ℃ in the dark for 2 days and then purified using a 21% maltose solution to remove the dead microspores. The surviving microspores were collected by centrifugation, washed twice with the induction medium, and then adjusted to a density of 1.0 × 10^5^ microspores/mL. One milliliter of the microspore suspension was inoculated in a Petri dish (35 × 12 mm, NEST Biotechnology, Wuxi, China), sealed with Parafilm (Parafilm® M, Bemis, Neenah, WI, USA), and incubated in the dark at 25 ℃ for 23 days until callus formation, with 3 replicates for each sample.

### Optimization of induction medium on callus initiation

The microspores were treated with different induction media (IM). The eight IMs composed by two basic media (N6 and N6 macronutrients with B5 micronutrients and vitamins (NB)), two carbon sources (maltose and sucrose), and two medium formulations (MF1 and MF2), as shown in Table 1. All the IMs were adjusted to pH 5.8 and sterilized by filter-sterilization (0.22 μm PES). N6 and NB were purchased from PhytoTech Labs (Kansas, USA).

**Table 1:** List of different induction media

Medium formulation	Basic medium	Carbon source	Other components	Number of Induction medium
MF1	N6	Maltose (75 g/L)	Proline (0.5 g/L), Glutamine (0.5 g/L) 2.4-D (2 mg/L) MES (0.976 g/L)	IM7
	NB			IM6
	N6	Sucrose (60 g/L)		IM5
	NB			IM2
MF2	N6	Maltose (75 g/L)	Glutamine (0.75 g/L) 2.4-D (2 mg/L) KT (0.5 mg/L) MES (0.976 g/L)	IM1
	NB			IM3
	N6	Sucrose (60 g/L)		IM4
	NB			IM8

2,4-D: 2,4-Dichlorophenoxyacetic Acid; KT: kinetin

### Effects of plant growth regulators on callus initiation

The isolated microspores were subjected to different combinations of 2,4-D (0.5, 1, and 2 mg/L) and KT (0, 1.5, and 3.0 mg/L) on N6 basal medium supplemented with 60 g/L sucrose, 0.5 g/L proline, 0.5 g/L glutamine, and 0.976 g/L MES for callus initiation, with 3 replicates for each sample. The nine media were adjusted to pH 5.8 and sterilized by filter-sterilization (0.22 μm PES).

### Plantlet regeneration

Calli from different genotypes were incubated in differentiation media (DM) to produce green shoots. Two differentiation media (DM1 and DM2) were used for the calli from NJ46 and CXJ, while calli from Nipponbare and ZH11 were cultured on DM1 only. The differentiation media were renewed once per month over a period of 2–3 months. The green shoots were then transferred to the rooting medium for one month to facilitate the formation of complete plantlets.

The DM1 contained 1/2 MS (PhytoTech, Kansas, USA), 2 mg/L 6-benzylaminopurine (6-BA), 0.5 mg/L 1-naphthaleneacetic acid (NAA), 30 g/L sucrose, solidified with 6 g/L agar (Solarbio, Beijing, China). The DM2 contained 1/2 MS, 2 mg/L KT, 0.5 mg/L NAA, 1 mg/L 6-BA, 30 g/L maltose, solidified with 6 g/L agar. The rooting medium contained 1/2 MS, 0.05 mg/L NAA, and 2 mg/L chlormequat chloride (CCC), 30 g/L sucrose, solidified with 6 g/L agar. These media were adjusted to pH 5.8 and then sterilized by autoclave sterilization (IMJ-78A, STIK, Shanghai, China) at 121 ℃, 0.11 Mpa for 20 min.

### Fertility evaluation

The regeneration plantlets from the isolated microspore culture were removed from the rooting medium, washed well, and secured with sponge strips on perforated foam boards. These boards floated in a plastic container (52 × 35 × 15 cm) with 10 L of Hoagland solution (Coolaber, Beijing, China). The solution was renewed every seven days and aerated continuously using an electric pump. All plantlets were placed in the greenhouse (day/night temperature, 28/22 °C; light/dark, 16/8 h; 60% relative humidity) under approximately 400 μmol photon m^−2^ s^−1^ light intensity. After about five months, some plants had seeds. The numbers of fertile and sterile plants were counted.

### Statistical analyses

The callus yield was determined by weighing the total calli formed in each petri dish after 23 days of induction. The differentiation frequency was defined as the number of green plantlets formed from each 100 mg calli. All the experimental data were analyzed by one-way analysis of variance and the least significant difference (LSD) tests using DPS software (version 7.05, China). Different letters after the mean values or on the bar indicate statistically significant differences in the same treatment at *P* < 0.05.

## Results

### Rice isolated microspore culture

**Fig. 2:**
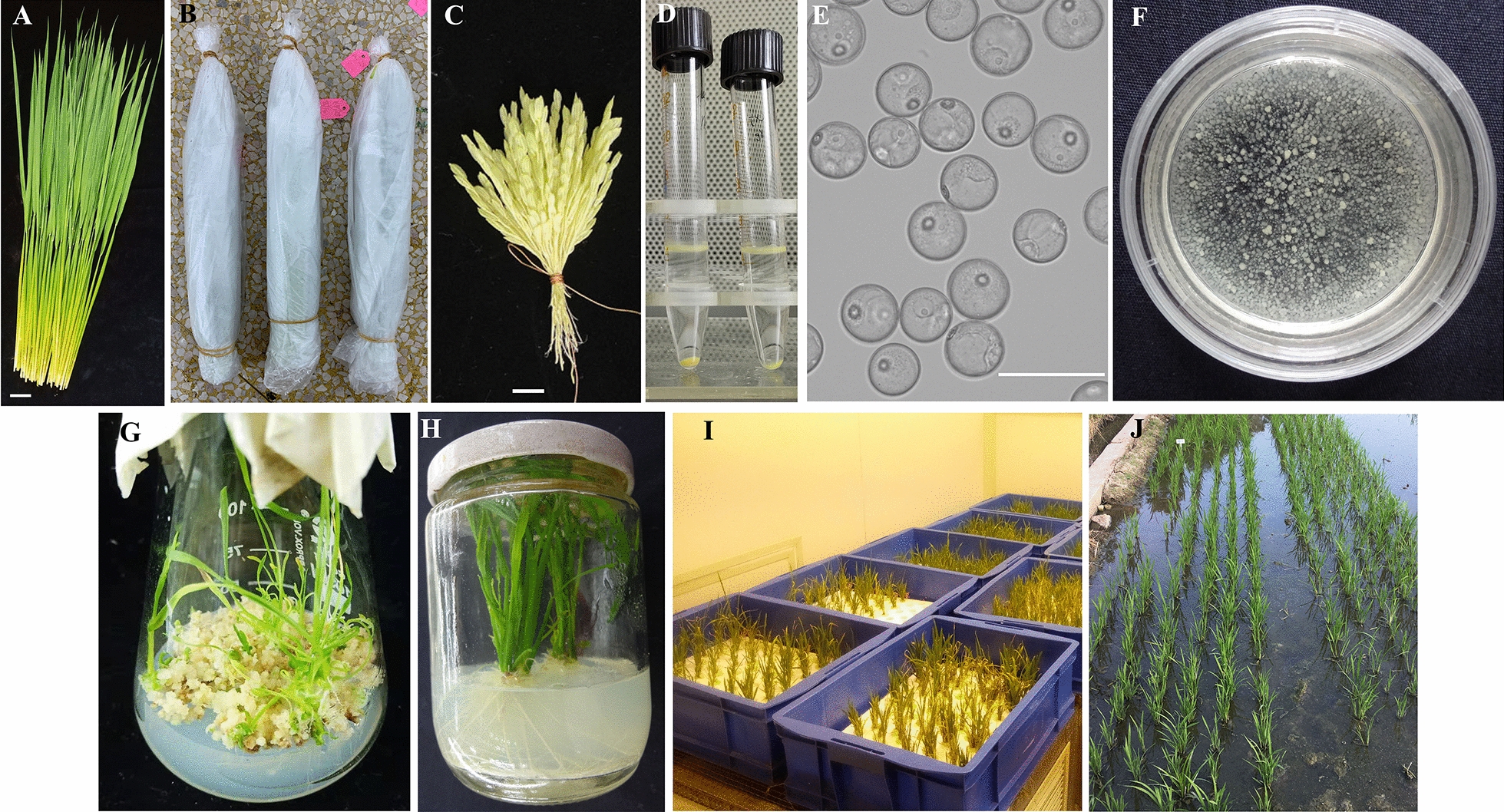
Rice isolated microspore culture. **A** Young panicle collection, bar = 5 cm, **B** Panicle wrapped for low-temperature pre-treatment, **C** Primary branch selection, bar = 1 cm, **D** Microspore collection and purification, **E** Isolated microspores in induction medium, bar = 75 μm, **F** Callus induction from the isolated microspores, **G** Green shoots differentiation from calli, **H** Well-rooted green plantlets, **I** Hydroponic cultivation in greenhouse, **J** Field-planting of microspore- derived plantlets

The isolated microspore culture process in rice consisted of the following key steps (Fig. 2). Five of these key steps were optimized, namely, the duration of low-temperature pre-treatment (B), selection of the microspore developmental stage (C), optimization of the induction medium and plant growth regulators (PGRs) (E and F), and optimization of the differentiation medium (G).

### Effects of the durations of low-temperature pre-treatment on callus induction

**Fig. 3:**
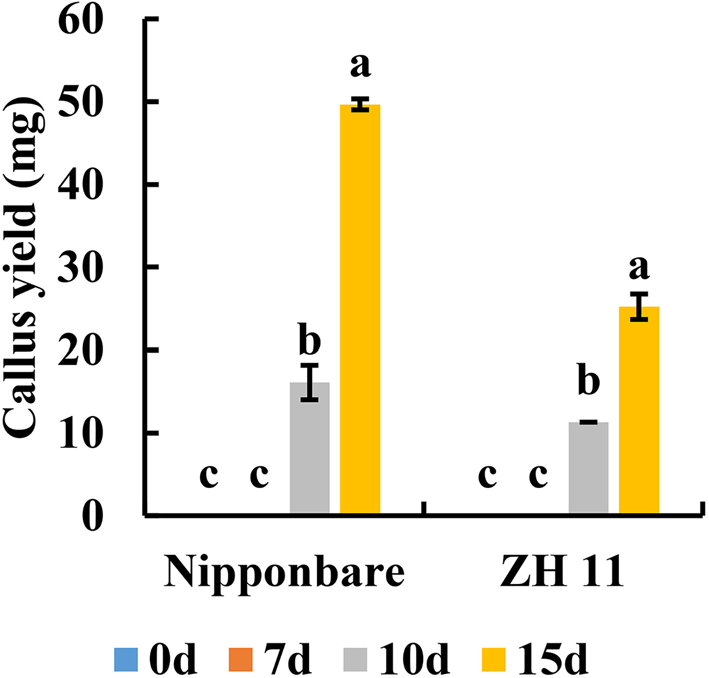
Effects of the durations of low-temperature pre-treatment on callus induction in Nipponbare and ZH11. Data are presented as mean ± standard deviation (SD) (n = 3). Different lower-case letters on bars indicate statistically significant intergroup differences (*P* < 0.05)

The effects of different low-temperature pre-treatment durations on callus induction were assessed in Nipponbare and ZH11. The microspores isolated from young panicles exposed to different durations of low-temperature pre-treatment were cultured on IM5 medium. The results are shown in Fig. 3. The callus yield was found to increase with the extension of low-temperature pre-treatment duration. The calli were generated only after 10 days of cold pre-treatment, and the callus yield after 15 days of pre-treatment was more than twice that after 10 days of pre-treatment. When the low-temperature pre-treatment exceeded 15 days, the young panicle was in a poor condition and particularly easy to cause contamination during the isolated microspore culture. Therefore, the optimal duration of the low-temperature pre-treatment was between 10 and 15 days.

### Primary branch classification and microspore developmental stage

**Fig. 4:**
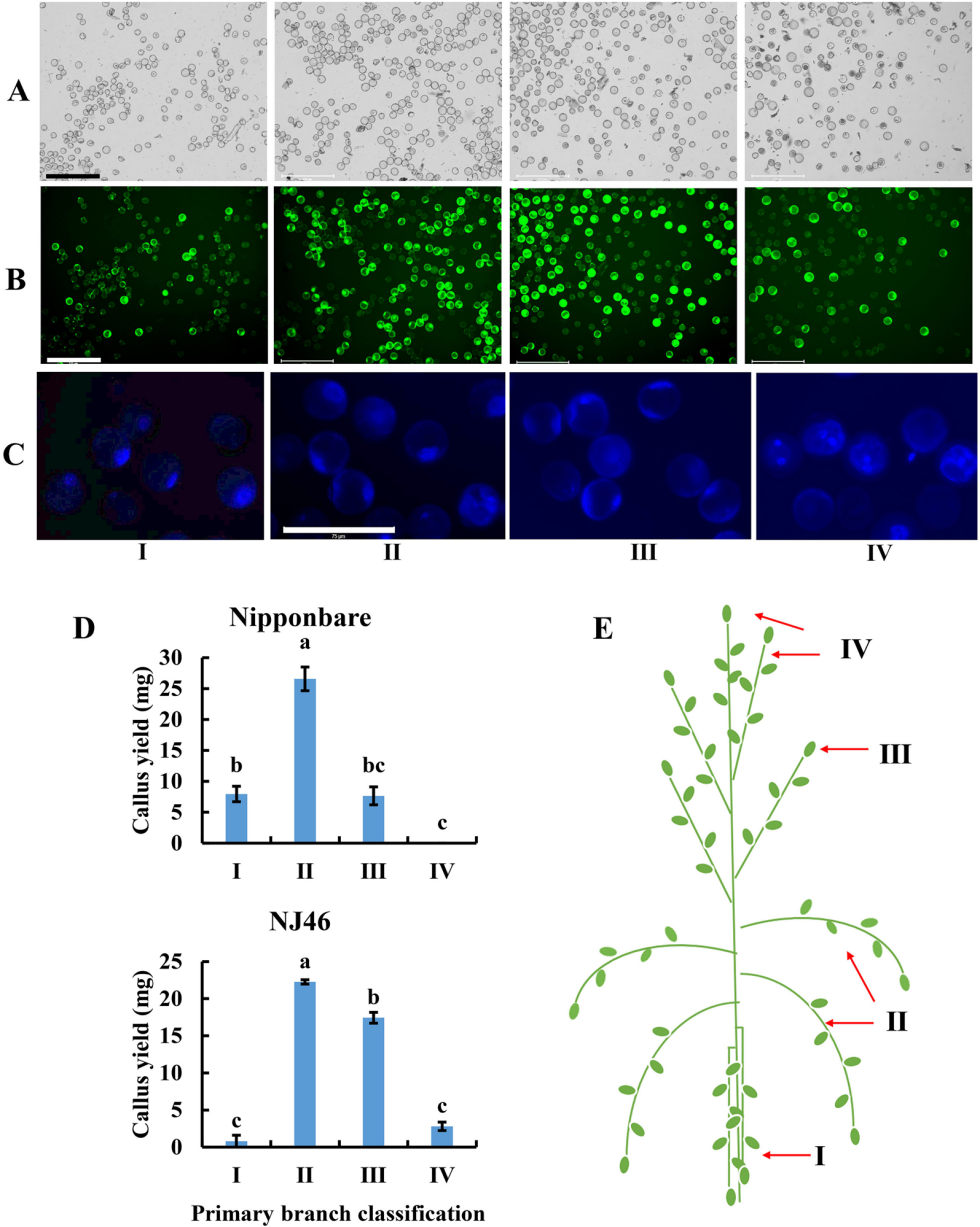
Microspore viability, developmental stage, callus yield, and young panicle diagram of different primary branch classifications. **A** Microspore in bright field; All microspores can be observed; bar = 275 μm; **B** Microspore in dark field; High and dense fluorescence indicated the viable microspores; bar = 275 μm; **C** Different microspore developmental stages; bar = 75 μm. **D** Callus yields from different primary branch classifications of Nipponbare and NJ46. Data are presented as mean ± SD (n = 3). Different lower-case letters on bars indicate the statistically significant differences (*P* < 0.05). **E** A simple and convenient sampling model. The optimal stage for isolated microspore culture is Type II

The relationship between the developmental stage of microspores and the curvature of the primary branches with the color of the florets was investigated in Nipponbare (Table 2 and Fig. 4A, B, C). The results showed that the microspores from type II had the highest viability rate (54.70%), and the developmental stage of these viable microspores was in the mid-late uninucleate stage (85.98%) and early binucleate stage (14.02%). The microspores from these four types were then cultured on IM5 medium for callus induction. It was found that the callus yield from type II was the highest. This classification method was verified in NJ46 using the same sampling criteria, with similar results (Fig. 4D). These results indicated that the microspore developmental stage could be predicted according to the curvature of the primary branch and the color of the floret. This led to the proposal of a simple and convenient sampling model (Fig. 4E). After low-temperature pre-treatment, the different types of branches could be clearly distinguished by holding the top branch. The second type of primary branch was retained and excess florets were removed according to their color. The microspores in the remaining florets were the optimal stage for isolated microspore culture.

**Table 2:** Microspore viability and developmental stage of different primary branch classifications

Classification of primary branch	Microspore viability (%)	Viable microspore developmental stage	
		Uninucleate (%)	Binucleate (%)
Type I	10.91 ± 0.64^d^	100.00 ± 0.00^a^	0.00 ± 0.00^d^
Type II	54.70 ± 1.40^a^	85.98 ± 1.38^b^	14.02 ± 1.38^c^
Type III	46.13 ± 1.38^b^	56.79 ± 1.61^c^	43.21 ± 1.61^b^
Type IV	30.71 ± 1.05^c^	8.33 ± 0.63^d^	91.67 ± 0.63^a^

Data are presented as mean ± SD (n = 3). Different superscript lower-case letters after the mean values indicate statistically significant differences (*P* < 0.05)

### Effects of different induction media on callus induction in different genotypes

**Fig. 5:**
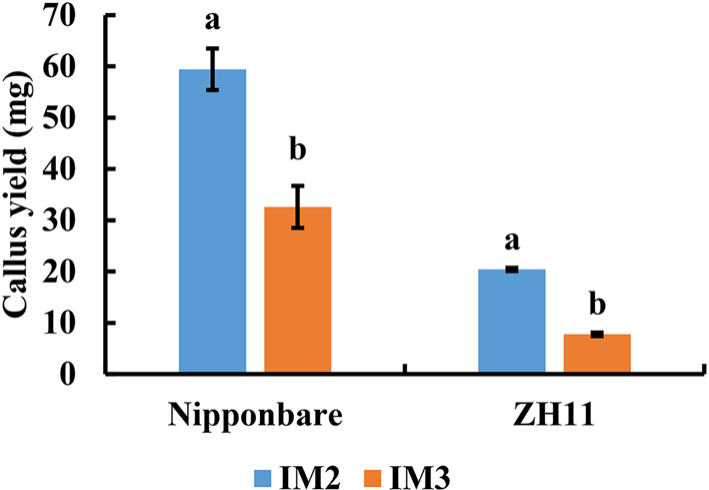
Callus yields of isolated microspore culture from IM2 and IM3 of Nipponbare and ZH11. Data are presented as mean ± SD (n = 3). Different lower-case letters on bars indicate statistically significant intergroup differences (*P* < 0.05)

The influence of different induction media on callus induction by isolated microspore culture from NJ46 and CXJ were explored and the results are shown in Table 3. The composition of MF1 was found to be more suitable for isolated microspore culture in NJ46, among which IM2 has the best culture effect followed by IM6. The basic NB medium was observed to be more suitable for isolated microspore culture in CXJ, with IM3 medium resulting in the highest callus yield. The effect of the IM2 medium did not differ significantly from that of IM3, suggesting its used as a viable alternative medium for isolated microspore culture in CXJ. The effects of IM2 and IM3 media were then tested on Nipponbare and ZH11. The results showed that the callus yield on the IM2 medium was significantly higher than that on the IM3 medium for both Nipponbare and ZH11 (Fig. 5).

**Table 3:** Effects of different induction media on callus induction in different genotypes

Medium formulation	Basic medium	Carbon source	Number of induction medium	Callus yield (mg)	
				NJ46	CXJ
MF1	N6	Maltose	IM7	97.15 ± 19.95^c^	3.85 ± 0.55^e^
	NB		IM6	151.30 ± 16.40^b^	82.90 ± 9.40^b^
	N6	Sucrose	IM5	91.60 ± 8.00^c^	42.80 ± 2.80^ cd^
	NB		IM2	223.35 ± 16.15^a^	124.00 ± 1.40^a^
MF2	N6	Maltose	IM1	60.10 ± 7.40^ cd^	24.30 ± 1.10d^e^
	NB		IM3	87.20 ± 1.10^c^	130.15 ± 11.75^a^
	N6	Sucrose	IM4	0.00 ± 0.00^e^	8.00 ± 8.00^e^
	NB		IM8	27.00 ± 2.20^de^	63.05 ± 4.35^bc^

Data are presented as mean ± SD (n = 3). Different superscript lower-case letters after the mean values indicate statistically significant differences (*P* < 0.05)

### Effects of plant growth regulators on callus induction in different genotypes

The effects of 2,4-D and KT concentrations on callus induction were evaluated (Table 4). The results indicated that the highest callus yield was produced using 1.0 mg/L 2,4-D and 0 mg/L KT. The addition of 1.5 mg/L KT reduced the callus yield to between 65 and 85% of the yield without the addition of KT. Almost no callus was generated at the concentration of 3.0 mg/L KT. These results suggested that callus generation was significantly affected by the high concentrations of KT.

**Table 4:** Effects of 2,4-D and kinetin on callus induction in NJ46 and CXJ

2,4-D (mg/L)	KT (mg/L)	Callus yield (mg)	
		NJ46	CXJ
0.50	0.0	56.10 ± 2.75^c^	124.00 ± 4.92^b^
	1.5	12.93 ± 0.33^d^	36.3 ± 2.89^c^
	3.0	0.00 ± 0.00^e^	0.00 ± 0.00^d^
1.00	0.0	76.23 ± 4.45^a^	166.93 ± 7.53^a^
	1.5	11.27 ± 3.96^d^	49.20 ± 5.37^c^
	3.0	0.00 ± 0.00^e^	2.20 ± 1.59^d^
2.00	0.0	66.10 ± 6.12^b^	135.50 ± 11.41^b^
	1.5	16.8 ± 3.26^d^	46.97 ± 3.96^c^
	3.0	1.43 ± 1.43^e^	0.00 ± 0.00^d^

Data are presented as mean ± SD (n = 3). Different superscript lower-case letters after the mean values indicate statistically significant differences (*P* < 0.05)

### Optimization of the differentiation medium in different genotypes

**Fig. 6:**
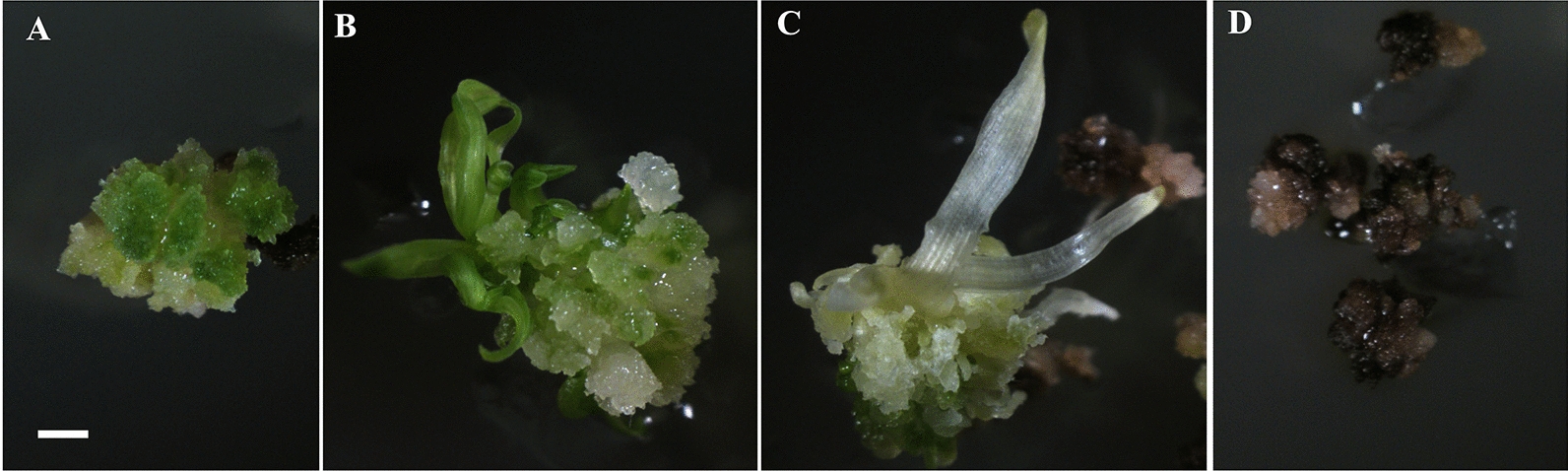
Shoot regeneration from microspore calli. **A** Green spots initiation, **B** Green shoot developed from microspore callus, **C** Albino shoot developed from microspore callus, **D** Callus browning, bar = 1 cm

The calli generated by isolated microspores culture were transferred to differentiation media for the green shoot regeneration. After 3 to 4 weeks of culture on the regeneration medium, green spots were observed on the surfaces of some calli (Fig. 6A). Subsequently, the calli with green spots developed into green shoots (Fig. 6B), albino shoots (Fig. 6C), or browning (Fig. 6D).

**Fig. 7:**
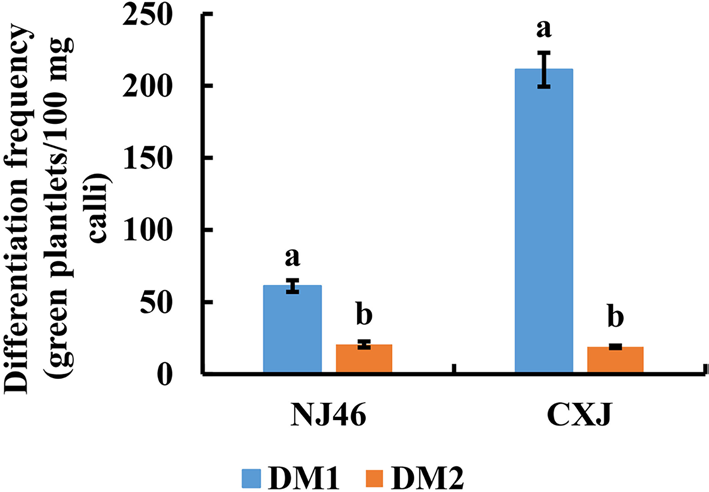
Comparison of differentiation frequency of NJ46 and CXJ on two regeneration media. Data are presented as mean ± SD (n = 3). Different lowercase letters on bars indicate statistically significant intergroup differences (*P* < 0.05)

The effects of two differentiation media (DM1 and DM2) were assessed using calli from NJ46 and CXJ. it was found that the differentiation frequencies on DM1 were significantly higher than those on DM2 for both NJ46 and CXJ (Fig. 7). On DM1, the differentiation frequencies of NJ46 and CXJ were 61 and 211 green plantlets per 100 mg calli respectively. On DM2, the differentiation frequencies of NJ46 and CXJ were 21 and 19 green plantlets per 100 mg calli, respectively. The calli from ZH11 and Nipponbare were only cultured on DM1, resulting in the differentiation frequencies of 73 and 63 green plantlets per 100 mg calli, respectively.

### Investigation of the fertility of regenerated plantlets

The regenerated green plantlets were grown hydroponically in the greenhouse. Spontaneous DH plants can produce seeds, while haploid plants are infertile. The fertility of the regenerated green plantlets from NJ46 and CXJ was investigated. Of 115 regenerated green plantlets from NJ46, 39 were sterile and 76 were fertile, with an estimated double haploid frequency of 66.09%. Of 128 regenerated green plantlets from CXJ, 93 were sterile, and 35 were fertile, with an estimated double haploid frequency of 27.34%. This data imply that the frequency of chromosomal doubling in the microspore-derived plants was influenced by the genotype.

## Discussion

DH techniques can be effectively integrated with other biotechnological approaches to produce innovative breeding outcomes, and they offer extensive potential for improving crop agronomic traits. They also can be widely used in genetic research. [19]. The development of an effective system of isolated microspore culture is essential for rice improvement through biotechnology, given the ability of isolated microspore culture to produce DHs.

Cold pre-treatment is commonly used as a stressor for the promotion of androgenesis in rice and other cereals [25]. The low-temperature treatment induces and ensures the continued development of the sporophyte mode of microspore rather than gamete formation [20]. The beneficial effects of cold pre-treatment on callus induction mainly include the delay of anther wall senescence, the promotion of symmetrical pollen division, and the release of substances required for androgenesis [23]. It has been reported that the temperature and treatment duration vary depending on the rice variety. Young panicles were generally treated with a range of temperatures from 4 to 10 ℃ for 3 to 28 d [15, 25, 26]. The present study investigated the effects of cold pre-treatment on microspore embryogenesis in the Nipponbare and ZH11 varieties. The results showed that cold pre-treatment at 4 ℃ for 10–15 d positively influence the initiation of microspore embryogenesis.

The developmental stage of the microspores is critical in androgenesis [17]. Only a specific microspore developmental stage is affected by induction treatment, and even within a single anther, only a certain percentage of microspores will switch from their normal gametophytic pathway to the sporophytic pathway [27]. The mid-late uninucleate stage is the most favorable stage for the in *vitro* induction of rice [23, 28]. Therefore, rapid and accurate identification of the microspore developmental stage before culture is vital for isolated microspore culture in rice. The cytological examination of the microspore's developmental stage is complicated and instrument-dependent. An easily observable morphological feature in rice that correlates well with the microspore developmental stage can be used as a marker for the identification of the required stage of microspore [28]. The distance between the nodes of the last two leaves has been used as a morphological index for the accurate determination of microspore maturity [20, 28]. Several factors influence the internodal distance, including the cultivar, plant height, and growth condition. Therefore, utilizing a standard internodal distance for assessing the microspore developmental stage in different rice varieties or the same rice variety grown under different conditions is not suitable [22]. Here, we provide a more precise morphological indicator to determine the developmental stage of microspores that is suited for isolated microspore culture in rice, as shown in Figs. 1 and 4. The degree of primary branch curvature is a simple morphological indicator that can be used to differentiate microspores at various stages, independent of the genotype and environmental influences. Most of the microspores in the yellow-green florets on the type II of the primary branch are in the mid-late uninucleate stage, the most suitable stage for isolated microspore culture in rice.

The culture medium plays an important role in androgenesis in rice, not only providing nutrition for microspores but also deciding their fate [23]. N6 medium is the most widely used basic medium for androgenesis [29]. Several modifications to the basal N6 medium have been explored for their effects on androgenesis [28, 30]. Kumar et al. [25] suggested that a modified N6 medium (N6_M_) could effectively activate recalcitrant genes and substitute for N6 medium. Tajedini et al. [31] proposed that B5 induction medium was the most appropriate for microspore embryogenesis in all studied rice cultivars. Carbon source is essential for androgenesis due to both osmotic and nutritional effects [32]. Previous studies have shown that maltose was superior to sucrose as the carbon source in anther culture of rice for callus induction [33, 34]. However, sucrose was still used as a carbon source for *indica* rice microspore culture [15]. Here, the effects of eight different induction media were evaluated on two distinct genotypes. The results indicated that the NB basal medium was effective in inducing callus formation, especially in CXJ. The composition of media based on NB medium supplemented with amino acids and 2,4-D was shown to be more appropriate for NJ46 microspore embryogenesis. Based on the results, two induction media, IM2 and IM3, were selected for callus induction in isolated microspore culture of other rice genotypes. These data also suggested that particular genotypes may have specific requirements in terms of the culture medium.

PGRs are important in microspore embryogenesis [31]. The optimal concentrations and ratios of auxin and cytokinin play major roles in early microspore embryogenesis processes [35]. 2,4-D is a commonly used PGR for the induction of embryogenic callus. It has been reported that the application of 0.5 mg/L 2,4-D and 3.0 mg/L KT could significantly enhance the callus initiation rate in the Malaysian *indica* rice variety MR219 [15]. However, our results indicated that high concentrations of KT (1.5—3.0 mg/L) significantly impeded callus formation. It is worth noting that the addition of 0.5 mg/L KT in IM3 medium resulted in the maximum callus yield in CXJ. Further investigation is needed to determine if adding lower concentrations of KT (less than or equal to 0.5 mg/L) can enhance callus initiation. Further optimization of the concentration of 2,4-D in the induction medium lead to maximum callus yield at a concentration of 1 mg/L. It is known that the callus produced under high 2,4-D levels had a low capacity for regeneration [16]. Therefore, it is important to consider the callus quality when enhancing the callus yield. In the present study, the concentration of 2, 4-D used in the induction medium was always 2 mg/L. It is necessary to reduce the concentration of 2, 4-D to 1 mg/L or even 0.5 mg/L to produce high-quality callus and obtain a higher regeneration frequency.

The two main stages in microspore embryogenesis, callus development from the microspore and green plantlet regeneration from the embryogenic callus, have different nutritional requirements and are therefore carried out on different culture media [28]. This study used calli from NJ46 and CXJ to assess the effects on differentiation of two differentiation media. The results demonstrated that DM1 was a better choice for callus differentiation from rice isolated microspore culture. For each 100 mg calli, the differentiation frequencies in the four genotypes varied from 61 to 211 green plantlets.

Under culture conditions, microspore-derived plants can spontaneously undergo chromosome doubling, occurring at frequencies up to 72% in rice [16]. According to research by Cha-um et al. [36], the spontaneous chromosome doubling frequency of regenerated plantlets from anther culture ranged from 21.5 to 31.9%. Lantos et al. [30] suggested that spontaneous chromosome doubling is influenced by the genotype. Based on the observation of fertile microspore-derived plants from two genotypes, we found that the estimated chromosome doubling frequencies of 66.09% and 27.34% respectively, indicating that there were genotypic differences in the frequency of chromosome doubling in isolated microspore culture. Further investigation into the influence of the genotype on the frequency of chromosome doubling in other genotypes is required. It is suggested that the ploidy levels of microspore-derived plantlets should be determined directly at an early stage by flow cytometry.

## Conclusion

In conclusion, an efficient *japonica* rice isolated microspore culture system was established. The duration of low-temperature pre-treatment, microspore developmental stage, induction medium, PGR concentration, and differentiation medium were optimized for callus initiation and green plantlet regeneration. In *japonica* rice, microspore embryogenesis was found to be effectively promoted by the application of low-temperature pre-treatment at 4 ℃ for 10–15 days. A simple and efficient method for identifying the microspore developmental stage was presented, and it was shown that the best stage for isolated microspore culture was when the florets on the primary branch of the second type (the primary branch curvature between 90 and 180 degrees) in the rice panicle were yellow-green. The IM2 induction medium (NB basic medium containing 60 g/L sucrose, 0.5 g/L proline, 0.5 g/L glutamine, 2 mg/L 2.4-D, and 0.976 g/L MES) or IM3 (NB basic medium containing 75 g/L maltose, 0.75 g/L glutamine, 2 mg/L 2.4-D, 0.5 mg/L KT, and 0.976 g/L MES) was optimal for callus induction, depending on the genotype. The optimal concentration of 2, 4-D in callus induction medium was 1 mg/L. Callus induction was negatively affected by concentrations of KT over 1.5 mg/L. DM1 medium composed of 1/2 MS, 2 mg/L 6-BA, 0.5 mg/L NAA, 30 g/L sucrose and 6 g/L agar was found to be an appropriate differentiation medium for the microspore callus of *japonica* rice. The regeneration frequency varied considerably among the four genotypes,, with CXJ having the highest regeneration frequency at 211 green plantlets per 100 mg calli.

## Data Availability

The datasets supporting the conclusions of this article are included within the article.

## Abbreviations

NJ46: Nanjing46
CXJ: Chongxiangjing
ZH11: Zhonghua11
MES: 2-(N-morpholino) ethane sulfonic acid hydrate
FDA: Fluorescein Diacetate
DAPI: 4',6-Diamidino-2-phenylindole
N6: Chu's N-6 Medium
B5: Gamborg’s B5
2,4-D: 2,4-Dichlorophenoxyacetic Acid
KT: Kinetin
DM: Differentiation medium
6-BA: 6-Benzylaminopurine
NAA: 1-Naphthaleneacetic acid
MS: Murashige and Skoog
CCC: Chlormequat chloride
LSD: Least significant difference. PGR: plant growth regulator
